# Cognitive–behavioral factors in tinnitus-related insomnia

**DOI:** 10.3389/fpsyg.2023.983130

**Published:** 2023-03-17

**Authors:** Gemma Barry, Elizabeth Marks

**Affiliations:** Department of Psychology, University of Bath, Bath, United Kingdom

**Keywords:** tinnitus, insomnia, CBT, cognitive, behavioral, sleep, sleep disorder

## Abstract

**Background:**

A significant proportion of individuals with distressing tinnitus also report insomnia. Limited, but emerging, evidence suggests that tinnitus-related insomnia cannot be explained only by the presence of tinnitus and that sleep-related cognitive–behavioral processes may play a key role in exacerbating tinnitus-related insomnia.

**Objectives:**

This study aimed to assess whether sleep-related cognitions and behaviors believed to maintain insomnia disorder are present in individuals with tinnitus-related insomnia.

**Methods:**

This between-groups study recruited 180 participants online for four groups: tinnitus-related insomnia (*N* = 49), insomnia disorder without tinnitus (*N* = 34), tinnitus sufferers who are good sleepers (*N* = 38), and controls (*N* = 59). They completed questionnaires assessing insomnia severity, sleep-related cognitions and behaviors, sleep quality, anxiety, and depression. People with tinnitus completed a measure of tinnitus severity and rated the loudness of their tinnitus on a subjective measure.

**Results:**

Linear regression demonstrated that group significantly predicted sleep related thoughts and behaviors, and sleep quality. Pairwise comparisons showed that the tinnitus-related insomnia group had significantly greater insomnia-related thoughts and behaviors and significantly worse sleep quality than tinnitus-good sleepers. No differences were seen between the tinnitus-related insomnia and the insomnia groups. The tinnitus-related insomnia group had significantly higher depression, anxiety, and tinnitus distress than tinnitus-good sleepers.

**Conclusion:**

Findings suggest that tinnitus-related insomnia may be maintained by cognitive–behavioral processes similar to those found in insomnia disorder. Such processes are more important than tinnitus severity when understanding sleep disturbance. People with tinnitus-related insomnia may benefit from treatments such as cognitive–behavioral therapy for insomnia.

## 1. Introduction

Tinnitus is defined by the experience of hearing sound, commonly ringing or buzzing, in the absence of external stimuli (Beukes et al., 2017). Estimates vary, with some suggesting it may affect 30% of the population (McCormack et al., 2016). Common impacts of distressing tinnitus include emotional distress, anxiety (Laurikainen et al., 2000), difficulties in sleeping, relationships, work functioning, and concentration (Asnis et al., 2018). A systematic review of the co-occurrence of depression and tinnitus suggested a 33% prevalence of depression among people who suffer from tinnitus (Salazar et al., 2019). It is likely that this co-occurrence is bidirectional, for example, tinnitus fueling depression and depression, possibly making tinnitus harder to cope with, thus making tinnitus worse. A United Kingdom survey estimated societal costs of tinnitus at £2.7bn annually (Stockdale et al., 2017).

Those suffering from tinnitus commonly complain of sleep disturbance (Hébert et al., 2011; Miguel et al., 2014), though our understanding of the comorbidity rates of insomnia and tinnitus is incomplete. A review examining the relationship between the two disorders (Asnis et al., 2018) found that 15 of 16 studies included in the review used variable and often inadequate assessment techniques and criteria to define insomnia. Most reported a prevalence of insomnia above 40% in their tinnitus sample (Asnis et al., 2018). The remaining study used diagnostic assessment (Miguel et al., 2014) and reported an insomnia prevalence of 27%. This review led to predictions that individuals who suffer from both disorders will experience greater tinnitus distress than those with only tinnitus and highlighted that higher tinnitus-related distress is associated with greater levels of anxiety and depression. Research has found that men who experience insomnia associated with tinnitus have higher depression scores than women, indicating potential gender differences to consider in treatment (Richter et al., 2021).

Our understanding of why sleep disturbance is common in tinnitus is limited. One hypothesis suggests that insomnia in tinnitus can be understood through the association between noise and difficulty sleeping (Izuhara et al., 2013). Izuhara et al. (2013) suggested that sleep disturbance associated with tinnitus could be similar to residents in a noisy neighborhood experiencing difficulties falling asleep, suggesting that tinnitus volume alone maintains insomnia. However, other research has indicated that the interpretation of tinnitus causes greater distress than its volume alone (Basile et al., 2013). A further study focused on the relationship between the loudness of tinnitus and insomnia (Aazah and Moore, 2019). The researchers found that the relationship was mediated by depression, tinnitus handicap, and tinnitus annoyance as opposed to insomnia being directly related to the loudness of tinnitus. It remains unclear if the experience of tinnitus can be compared to an external sound, particularly as there are different types and possible causes of tinnitus.

Insomnia disorder is commonly comorbid with psychological disorders such as anxiety and depression (Harvey, 2001); however, evidence indicates that it is not just a “symptom” of other psychological disorders (Harvey, 2001). This is because it can be treated independently from comorbidities and can both precede and follow comorbidities. The cognitive model of insomnia (Harvey, 2002) outlines many processes that an individual experiences during the night and day which maintain insomnia. In summary, it proposes that insomnia arises from worry about sleep and its consequential impact on day-to-day life. This causes increased emotional and physiological arousal, leading to selective attention and monitoring and distorted perceptions (i.e., underestimating sleep and functioning). Worries also lead to safety-seeking behaviors intended to reduce insomnia and its impact (e.g., canceling plans) but which paradoxically exacerbate sleep difficulties. This leads to escalating anxiety and further insomnia.

Cognitive–behavioral therapy for insomnia (CBTi) was developed as a brief psychological intervention for insomnia. Though it does not address all processes put forward in the cognitive model of insomnia (Harvey, 2002), it aims to target unhelpful thoughts and behaviors around sleep using psychoeducation, behavioral experiments, sleep restriction, stimulus control, and cognitive restructuring (Pigeon et al., 2012). Research shows that specifically targeting distorted perception of sleep leads to reduced sleep-related anxiety (Tang and Harvey, 2004), while challenging insomnia-related thoughts and behaviors enables individuals to establish healthier sleep patterns and improve their quality of life (Okajima et al., 2011). A high-quality meta-analysis of RCTs using CBTi in insomnia (Okajima et al., 2011) found it to be effective, with medium to large effect sizes. Improvement in measures of dysfunctional sleep-related cognitions was a significant change following CBTi, suggesting these are central to maintaining insomnia and crucial to target in treatment.

It is argued that the same cognitive and behavioral processes maintain insomnia, regardless of comorbidities such as chronic pain (Tang et al., 2012). CBTi is proven to successfully treat insomnia where such comorbidities exist (Jungquist et al., 2010). Of note, chronic pain shares many similarities with tinnitus (Moller, 2000), both being sensory perceptual disorders associated with hypersensitivity (Rauschecker et al., 2015) and related psychological difficulties (Rauschecker et al., 2015).

Evidence has shown that people with insomnia and tinnitus share similar sleep processes, including physiological characteristics of sleep disturbance (Burgos et al., 2005). Polysomnography (PSG), the “gold standard” objective assessment for sleep disorders, has shown that patients with tinnitus experience more awakenings and greater difficulty with sleep onset than healthy controls (Burgos et al., 2005). This finding is similar to that reported when comparing people with insomnia to healthy controls. A systematic review of studies of polysomnography applied to patients with tinnitus found that few have assessed tinnitus sufferers’ sleep using this technique, instead of opting for self-report questionnaires (Teixeira et al., 2018), highlighting the need for future studies to use polysomnography as an objective evaluation method.

Crönlein et al. (2016) assessed sleep and psychological difficulties in samples with and without tinnitus using the Regensburg Insomnia Scale (RIS). People with tinnitus reported more difficulties with and greater worries about sleep, supporting the theory that insomnia in tinnitus may be maintained by the insomnia process outlined above. However, this study is limited by using the RIS, a brief outcome measure rather than a tool designed to assess insomnia-specific cognitions and behaviors. It also did not offer a comparison with the insomnia group without tinnitus. The authors suggested that anxiety and depression could be confounding factors that were not accounted for in their study.

Few tinnitus studies include measures of improved sleep as a primary or secondary outcome, and few consider the severity of sleep impairment in recruitment or analysis (Hesser et al., 2011). However, a clinic-based study evaluated outcomes from CBTi for tinnitus-related insomnia (Marks et al., 2019) and found that 66.7% of participants showed reliable improvement on the Insomnia Severity Index (ISI). Participants reported reduced tinnitus severity and psychological distress. Though limited by the small sample size and lack of a control group, it was the first of its kind to provide evidence of the efficacy of CBTi as a treatment for tinnitus-related insomnia. A more recent randomized controlled study looked at the benefits of CBTi for people with tinnitus-related insomnia and found it to be more effective in reducing tinnitus distress and improving sleep quality than audiology-based care or a sleep support group (Marks et al., 2022). These studies offer tentative evidence that tinnitus-related insomnia is maintained by cognitive and behavioral processes similar to those seen in insomnia without comorbidities or insomnia disorder (Harvey, 2002).

In summary, evidence shows that the physiological characteristics of insomnia are similar in populations with and without tinnitus (Burgos et al., 2005), both experiencing decreased sleep efficiency, sleep time, and a higher number of awakenings than healthy controls (Burgos et al., 2005). Limited evidence indicates that the psychological processes that may underpin the experience of insomnia in tinnitus could be equivalent to the psychological processes that underpin insomnia without tinnitus (Crönlein et al., 2016; Marks et al., 2019). An example of this would be dysfunctional sleep-related cognitions, which are beliefs about people’s expectations and attitudes about the causes and consequences of poor sleep, e.g., “when I sleep poorly one night, I know it will disturb my sleep schedule for the whole week.” Our understanding in this area is hampered by a lack of consistency across the field regarding the outcome measures used to assess sleep quality, the presence of insomnia, and sleep-related thoughts and behaviors. It is vital to clarify how psychological factors are associated with tinnitus-related insomnia to improve understanding of patient experiences and effective treatment of tinnitus-related insomnia.

This study aimed to examine whether cognitive and behavioral factors believed to commonly maintain insomnia (Harvey, 2002) are equally important factors that contribute to insomnia in people who have tinnitus. It was hypothesized that levels of dysfunctional sleep-related cognitions, behaviors, and sleep quality would be similar in individuals reporting insomnia, regardless of tinnitus presence, and greater than in people who do not report insomnia (both with and without distressing tinnitus). As research has suggested that those with tinnitus and insomnia experience higher levels of anxiety, depression, and tinnitus distress than individuals who have tinnitus without insomnia (Asnis et al., 2018), it was hypothesized that tinnitus distress, anxiety, and depression would be significantly higher in tinnitus-related insomnia than in tinnitus-good sleepers. A final hypothesis was that subjective tinnitus volume would not differ significantly between the tinnitus-related insomnia group and the tinnitus-good sleeper’s group to add weight to the argument that it is not the noise of the tinnitus that keeps people who have insomnia awake.

## 2. Materials and methods

### 2.1. Design

This cross-sectional study used a between-groups design in which four groups were compared: (1) tinnitus-related insomnia, (2) insomnia, (3) tinnitus-good sleepers, and (4) controls. All responses were collected *via* the online platform Qualtrics; questionnaires were presented in the same order to participants. The inclusion criteria were a minimum age of 18. Exclusion criteria were existing diagnoses of a sleep disorder other than insomnia, or the belief that their insomnia was explained by a physical or mental health condition in addition to tinnitus. Recruitment was conducted *via* social media, with relevant charities agreeing to share the study on their websites.

### 2.2. Participants and procedures

A total of 773 participants initiated the study, of which 266 did not consent, and 73 exited the survey before completion. Significantly more (*N* = 253) met the criteria for the controls and the tinnitus-related insomnia group than planned and were prevented from participating. One participant was excluded as they wrote “333” in all free-text boxes. The final sample consisted of 180 participants who had completed all questionnaires (all but the GAD-7 and PHQ-9 were counted as complete data sets). Participants were asked whether or not they had tinnitus and a further question to assess how much of a problem it was for them. Participants then completed The Insomnia Severity Index (ISI; Morin, 1993). This information was then used to sort participants into one of the four groups (Supplementary Figure 1 in Supplementary material shows the study flow). The final groups were as follows: insomnia (*N* = 34); tinnitus-related insomnia (*N* = 49); tinnitus-good sleepers (*N* = 38); and controls (*N* = 59). See Table 1 for the demographic characteristics of the sample.

**Table 1:** Demographic characteristics of participants in the study exploring cognitive–behavioral factors in tinnitus-related insomnia.

Demographics	Insomnia	Tinnitus-related insomnia	Tinnitus-good sleepers	Controls	Value of *p*
Age (*N* = 180) *M* (SD)	34.97^b^ (15.29)	48.86^a^ (11.39)	43.08^a^ (16.27)	41.53^b^ (14.19)	<0.001
**Gender (*N* = 178)**					
Male (*N* = 65)	13 (38.2)	21 (42.9)	17 (44.7)	14 (24.6)	0.14
Female (*N* = 113)	21 (61.8)	28 (57.1)	21 (55.3)	43 (75.4)	
**Marital Status (*N* = 176)**					
Married/living with partner (*N* = 102)	13 (38.2)	25 (52.1)	24 (63.2)	40 (71.4)	0.013
Not married/unpartnered (*N* = 74)	21 (61.8)	23 (47.9)	14 (36.8)	16 (28.6)	
**Ethnicity (*N* = 176)**					
White (*N* = 159)	25 (73.5)	47 (97.9)	35 (92.1)	52 (92.9)	0.002
Non-white (*N* = 17)	9 (26.5)	1 (2.1)	3 (7.9)	4 (7.1)	
**Education (*N* = 176)**					
Tertiary/further (*N* = 129)	22 (64.7)	34 (70.8)	29 (76.3)	44 (78.6)	0.49
Less than tertiary/other (*N* = 47)	12 (35.3)	14 (29.2)	9 (23.7)	12 (21.4)	
**Employment (*N* = 176)**					
Full-time (*N* = 87)	15 (44.1)	24 (50.0)	19 (50.0)	29 (51.8)	0.92
Not full-time (*N* = 89)	19 (55.9)	24 (50.0)	19 (50.0)	27 (48.2)	
**Shift/night work (*N* = 176)**					
Yes (*N* = 15)	3 (8.8)	6 (12.5)	4 (10.5)	2 (3.6)	0.40
No (*N* = 161)	31 (91.2)	42 (87.5)	34 (89.5)	54 (96.4)	

*p*-values are derived from chi-squared tests for categorical variables and *t*-tests for continuous variables. Values in cells are expressed as *n* (%) unless otherwise specified. a, b values sharing superscripts do not differ (*p* > 0.05).

### 2.3. Measurement

All participants completed the following self-report questionnaires.

#### 2.3.1. The insomnia severity index (ISI)

This seven-item measure was used to screen participants for insomnia (Morin, 1993). The measure uses a 5-point Likert scale, ranging from 0 to 4, and offers a clinically relevant tool for assessing insomnia. Participants were allocated to a certain group depending on their score on this measure. The ISI has good reliability and validity (Bastien et al., 2001). A score of 10 or above is deemed appropriate for identifying clinically relevant insomnia in a community sample (Morin et al., 2011). The ISI has been shown to have excellent internal consistency (Cronbach’s *α* = 0.75) when used to assess insomnia in a community sample (Morin et al., 2011).

#### 2.3.2. The Pittsburgh sleep quality index (PSQI)

This was used to assess participants’ sleep quality (Buysse et al., 1989). The 18-item measure produces seven component scores and one overall global score relating to the quality of sleep. Question 10 was omitted from the administration as it does not contribute to the PSQI global score. This measure has acceptable internal consistency (Cronbach’s *α* = 0.75; Hinz et al., 2017). Participants who entered time ranges, for example, answering 9–11 p.m. when asked what time they have usually gone to bed in the past month, were allocated a mid-point. This measure was used to assess sleep quality instead of the ISI as it includes both quantitative aspects of sleep, such as sleep duration, and more subjective constructs, such as “restfulness” (Buysse et al., 1989).

#### 2.3.3. Dysfunctional beliefs and attitudes about sleep (DBAS-16)

The DBAS-16 was used to assess participants’ sleep-related beliefs, many of which become the target of CBTi (Morin et al., 2007). Participants are asked to respond to statements relating to beliefs and attitudes about sleep on a Likert scale ranging from 0 (strongly disagree) to 10 (strongly agree). Examples of dysfunctional beliefs participants are asked to respond to are “without an adequate night’s sleep, I can hardly function the next day” and “I am worried that I may lose control over my sleep abilities.” The DBAS-16 has been shown to have acceptable validity and reliability (Cronbach’s *α* = 0.77–0.79; Morin et al., 2007).

#### 2.3.4. Sleep-related behaviors questionnaire (SRBQ)

This measures the extent to which an individual engages in sleep-related safety behaviors thought to maintain insomnia (Ree and Harvey, 2004). Participants respond to 32 statements about how often they engage in each behavior, rating from 0 (almost never) to 4 (almost always). Examples of sleep-related safety behaviors are “I catch up on sleep by napping” and “I try to stop all thinking when trying to sleep.” The psychometric properties of the SRBQ have not been systematically evaluated (Lebrun et al., 2020). It has been shown to have good sensitivity to detect a change in psychological therapy for insomnia (Harvey et al., 2007), so it is the best available measure. Cronbach’s *α* = 0.94 for this sample, indicating excellent internal consistency.

#### 2.3.5. The generalized anxiety disorder assessment (GAD-7)

This seven-item scale assesses anxiety symptoms over the past 2 weeks (Spitzer et al., 2006). Participants’ rate anxiety experiences from 0 (not at all) to 3 (nearly every day). Internal consistency of the GAD-7 is excellent (Cronbach’s *α* = 0.92) and good test–retest reliability (*α* = 0.83; Spitzer et al., 2006). A score between 11 and 15 reflects moderate anxiety, and above 15 indicates severe anxiety.

#### 2.3.6. The patient health questionnaire (PHQ-9)

This nine-item scale assesses depression symptoms over the past 2 weeks, with respondents rating how much they were bothered by symptoms from 0 (not at all) to 3 (nearly every day) (Kroenke et al., 2001). The scale has good internal consistency (Cronbach’s *α* = 0.89) and good test–retest reliability (*α* = 0.84). A score between 10 and 14 reflects moderate depression, 15 and 19 indicates moderately severe depression, and above 19 indicates severe depression.

#### 2.3.7. Demographic information

A variety of demographic information was collected, including age, gender, ethnicity, and shift work, as this is known to impact sleep and circadian rhythms (Boivin and Boudreau, 2014).

The following measures were *only* completed by those who identified as having tinnitus.

#### 2.3.8. Tinnitus handicap inventory (THI)

This 25-item measure was used to assess the severity of the impact of participants’ tinnitus on their daily lives (Newman et al., 1996). The THI requires participants to respond yes, no, or maybe to statements about their tinnitus. The THI has been found to have high internal consistency (Cronbach’s *α* = 0.92). This measure was chosen as it focuses less on sleep impairment than other tinnitus questionnaires, which reduced the risk of redundant questions.

#### 2.3.9. Tinnitus-related distress

This was a single question that asked participants to rate how much of a problem their tinnitus was. Respondents chose between “not a problem,” “minor problem,” “moderate problem,” “considerable problem” or “severe problem.” Categories were informed by research looking at categories of tinnitus-related distress (Handscomb, 2006). This, along with the ISI, was used to inform which group participants were sorted into. This was used instead of the THI to insure the tinnitus groups both contained participants with at least moderate distress.

#### 2.3.10. Tinnitus visual analogue scale (VAS)

This assesses the subjective loudness of tinnitus. Participants were selected from 0 (I cannot hear my tinnitus, even in quiet) to 100 (my tinnitus is louder than any other noise), where they would currently rate their tinnitus. A Cochrane Review of CBT for Tinnitus (Martinez-Devesa et al., 2010) found numeric visual analogue scales used in seven of the eight included studies. However, variation in the presentation was noted. Single-item ratings, though limited, have been found to be more reliable than tinnitus loudness matching (Hall et al., 2017); this was included in addition to the THI due to the literature suggesting tinnitus loudness may be independent of tinnitus distress.

### 2.4. Data analysis

Data were analyzed using IBM SPSS v.25. Differences in participant demographics and general psychological characteristics (PHQ-9 and GAD-7) were explored across participant groups using the one-way ANOVAs for continuous variables and the chi-squared tests for categorical variables. Differences in anxiety and depression were tested using a non-parametric Kruskal–Wallis *H*-test as both variables were non-normally distributed, and no sensible transformations were identified.

ANCOVAs were planned *a priori* to test for differences between groups for insomnia-related cognitions, behaviors, and sleep quality when controlling for depression and anxiety. However, the assumption of homogeneity of regression slopes was violated, so linear regression analysis was used to test for participant group differences (while controlling depression and anxiety). For each outcome, models were fitted in hierarchical blocks [block 1: age and gender (female vs. male); block 2: anxiety and depression; block 3: participant group (four levels)] to test the incremental contribution of each set of variables to improvements in model fit (change in *F*-statistic and *R*^2^). Ethnicity was excluded from all regression models as there were too few non-white participants to make meaningful inferences. Pairwise differences between groups were tested *post-hoc* from a full model, including all covariates, with a Bonferroni correction for multiple tests. Differences between tinnitus-related insomnia and tinnitus-good sleeper’s groups were explored using the *t*-tests and the Mann–Whitney *U*-tests depending on the distribution of the outcome.

## 3. Results

### 3.1. Participant demographics

Demographic characteristics are presented in Table 1. Given small cell sizes for some variables, responses were collapsed such that there were two categories for each characteristic (e.g., male vs. female and white vs. non-white people).

The tinnitus-related insomnia group was found to be significantly older than the insomnia and control groups (*F*(3, 176) = 6.59, *p* < 0.001). Significant differences were also observed for marital status, *χ*^2^(3, *N* = 176) = 10.7, *p* = 0.013, and ethnicity, *χ*^2^(3, *N* = 176) = 14.7, *p* = 0.002, while no differences were observed with regard to gender, education level, and current employment. Table 2 presents summary scores across all measures. A Kruskal–Wallis *H*-test showed significant differences between participant groups for both anxiety, *H*(3) = 39.60, *p* < 0.001, and depression, *H*(3) = 71.7, *p* < 0.001. The tinnitus-related insomnia and insomnia groups had significantly greater (*p* < 0.01 for all) levels of anxiety and depression than tinnitus-good sleepers and controls. The tinnitus-related insomnia group compared with the insomnia groups and the controls compared with the tinnitus-good sleepers’ groups did not differ significantly.

**Table 2:** Summary of measures completed across groups in a study exploring cognitive–behavioral factors in tinnitus-related insomnia.

	Insomnia group (*N* = 34)	Tinnitus-related insomnia group (*N* = 48)	Tinnitus-good sleepers’ groups (*N* = 37)	Controls (*N* = 55)
ISI-Mean (SD), range	15.68 (3.54), 10–23	16.63 (4.05), 10–25	4.39 (2.71), 0–9	4.37 (2.65), 0–9
DBAS-16 – Mean (SD), range	5.77 (1.48), 1.81–8.06	5.85 (1.51), 2.81–9.63	3.51 (1.52), 0.63–6.50	3.21 (1.48), 0.38–6.69
SRBQ – Mean (SD), range	64.74 (17.87), 27–91	55.24 (17.56), 12–86	30.87 (13.80), 4–59	33.75 (19.44), 0–83
PSQI – Mean (SD), range	11 (2.98), 5–16	10.67 (2.75), 4–17	4.58 (2.14), 2–11	5.07 (2.43), 1–11
GAD-7 – Mean (SD), range	10.71 (5.32), 0–21	9.50 (6.00), 0–21	5.62 (4.68), 0–17	4.79 (3.82), 0–19
PHQ-9 – Mean (SD), range	12.15 (5.92), 3–25	12.29 (6.79), 1–26	4.77 (4.77), 0–20	4.04 (4.14), 0–23
THI – Mean (SD), range	26.33 (13.59), 6–48	58.16 (22.95), 10–98	41.9 (21.62), 6–88	19.50 (16.55), 16–92
VAS – Mean (SD), range	41.17 (18.93), 11–66	70.51 (19.82), 25–100	60.45 (19.60), 15–100	43.61 (19.16), 1–74

ISI, Insomnia Severity Index; DBAS-16, Dysfunctional Beliefs and Attitudes About Sleep; SRBQ, Sleep-Related Behaviors Questionnaire; PSQI, The Pittsburg Sleep Quality Index; GAD-7, The Generalized Anxiety Disorder Assessment; PHQ-9, The Patient Health Questionnaire; THI, Tinnitus Handicap Inventory; VAS, Tinnitus Visual Analogue Scale.

After exclusions for missing data on the outcome or covariates, 174 participants were included in the regression analysis (34 in the insomnia group, 48 in the tinnitus-related insomnia group, 37 in the tinnitus-good sleeper’s group, and 55 controls; Table 3).

**Table 3:** *F*-statistics, *R*-squared, and change in *R*-squared values derived from hierarchical linear regression models predicting sleep-related cognitions (DBAS-16) / behaviors (SRBQ) / sleep quality (PSQI) from age, gender, anxiety, depression, and group.

Model	Block	Variables	*F*	df	Value of *p*	*R* ^2^	Δ *R*^2^
DBAS-16 (*n* = 172)	1	Age, gender	0.28	2	0.76	0.00	–
	2	Anxiety, Depression	64.3	2	<0.001	0.44	0.44
	3	Group	10.5	3	<0.001	0.53	0.09
SRBQ (*n* = 174)	1	Age, gender	0.78	2	0.17	0.02	–
	2	Anxiety, Depression	71.6	2	<0.001	0.47	0.45
	3	Group	9.56	3	<0.001	0.55	0.08
PSQI (*n* = 174)	1	Age, gender	0.08	2	0.93	0.00	–
	2	Anxiety, Depression	5.72	2	<0.001	0.47	0.47
	3	Group	10.43	3	<0.001	0.66	0.19

df, degrees of freedom. DBAS-16, Dysfunctional Beliefs and Attitudes About Sleep; SRBQ, Sleep-Related Behaviors Questionnaire; PSQI, The Pittsburg Sleep Quality Index.

The results of the pairwise differences between groups, which were tested *post-hoc* from a full model, can be seen in Table 4.

**Table 4:** Effect estimates, adjusted 95% confidence intervals, and adjusted and unadjusted *p*-values.

Model	Comparison	Estimate	95% CI	*P*_adj_	*P*_unadj_
DBAS-16	Tinnitus-Related Insomnia – Insomnia	−0.04	−0.88 to 0.80	1.00	0.90
	Tinnitus-Related Insomnia – Tinnitus-Good Sleepers	1.41	0.51–2.23	<0.001	<0.001
	Tinnitus-Related Insomnia– Controls	1.45	0.60–2.31	<0.001	<0.001
	Insomnia – Tinnitus-Good Sleepers	1.46	0.53–2.38	<0.001	<0.001
	Insomnia – Controls	1.49	0.62–2.37	<0.001	<0.001
	Tinnitus-Good Sleepers – Controls	0.04	−0.72 to 0.80	1.00	0.90
SRBQ	Tinnitus-Related Insomnia– Insomnia	−9.20	−18.60 to 0.21	0.06	0.01
	Tinnitus-Related Insomnia – Tinnitus-Good Sleepers	11.18	1.13–21.23	0.02	<0.001
	Tinnitus-Related Insomnia – Controls	6.90	−2.61 to 16.42	0.33	0.06
	Insomnia – Tinnitus-Good Sleepers	20.38	10.01–30.74	<0.001	<0.001
	Insomnia – Controls	16.10	6.31–25.89	<0.001	<0.001
	Tinnitus-Good Sleepers – Controls	−4.28	−12.77 to 4.22	1.00	0.18
PSQI	Tinnitus-Related Insomnia– Insomnia	−1.03	−2.52 to 0.45	0.40	0.07
	Tinnitus-Related Insomnia – Tinnitus-Good Sleepers	4.15	2.56–5.73	<0.001	<0.001
	Tinnitus-Related Insomnia– Controls	3.44	1.94–4.94	<0.001	<0.001
	Insomnia – Tinnitus-Good Sleepers	5.18	3.54–6.82	<0.001	<0.001
	Insomnia – Controls	4.47	2.93–6.02	<0.001	<0.001
	Tinnitus-Good Sleepers – Controls	−0.71	−2.05 to 0.62	0.97	0.16

Model is adjusted for age, gender, anxiety, and depression. *P*_adj_ is adjusted using the Bonferonni method; *P*_unadj_ is not corrected for multiple comparisons. DBAS-16, Dysfunctional Beliefs and Attitudes About Sleep; SRBQ, Sleep-Related Behaviors Questionnaire; PSQI, The Pittsburg Sleep Quality Index.

### 3.2. Sleep-related cognitions

Results from a hierarchical regression model of sleep-related cognitions (DBAS-16 score) showed that the addition of anxiety and depression scores to predict sleep-related cognitions (DBAS-16 score) led to a statistically significant increase in *R*^2^ (Δ*R*^2^ = 0.44), *F*(2, 170) = 64.3 *p* < 0.001, as did the addition of the group, Δ*R*^2^ = 0.09, *F*(3, 169) = 10.5 *p* < 0.001. *Post-hoc* pairwise comparisons showed that the tinnitus-related insomnia group scored significantly higher on sleep-related cognitions than the tinnitus-good sleepers, difference = 1.41, 95% CI [0.51, 2.23] and controls, difference = 1.45, 95% CI [0.60, 2.31]. The insomnia group scored significantly higher on sleep-related cognitions than the tinnitus-good sleepers, difference = 1.46, 95% CI [0.53, 2.38] and the controls, difference = 1.49, 95% CI [0.62, 2.37]. The insomnia group had higher sleep-related cognitions than the tinnitus-related insomnia group. However, this difference was not significant: difference = 0.04, 95% CI [−0.88, 0.80], nor was the difference between the tinnitus-good sleepers and controls, difference = 0.04, 95% CI [−0.72, 0.80].

### 3.3. Sleep-related behaviors

Results from a hierarchical regression model of sleep-related behaviors (SRBQ scores) showed that the addition of anxiety and depression led to a statistically significant increase in *R*^2^; Δ*R*^2^ = 0.45, *F*(2, 172) = 71.6, *p* < 0.001, as did the addition of participant group, Δ*R*^2^ = 0.08, *F*(3, 171) = 9.56, *p* < 0.001. *Post-hoc* pairwise comparisons showed that the insomnia group scored highest on the SRBQ than other groups, with the difference between group means being significant for the tinnitus-good sleepers, difference = 20.38, 95% CI [10.01, 30.74] and controls, difference = 16.10, 95% CI [6.31, 25.89]. Though the insomnia group scored higher on average than the tinnitus-related insomnia group, the difference between group means was not significant, difference = −9.20, 95% CI [−18.60, 0.21]. The tinnitus-related insomnia group scored significantly higher than the tinnitus-good sleepers, difference = 11.18, 95% CI [1.13, 21.23] but not the controls, difference = 6.90, 95% CI [−2.61, 16.42]. The tinnitus-good sleeper’s group scored lower than the controls for sleep-related behaviors, but this difference was not significant, −4.28, 95% CI [−12.77, 4.22].

### 3.4. Overall sleep quality

Results from a hierarchical regression model of overall sleep quality scores (PSQI score) showed that the addition of anxiety and depression led to a statistically significant increase in *R*^2^; ΔR = 0.47, *F*(2, 172) = 5.72, *p* < 0.001, as did the addition of participant group, Δ*R*^2^ = 0.19, *F*(3, 171) = 10.43, *p* < 0.001. *Post-hoc* pairwise comparisons showed that the tinnitus-related insomnia group had significantly worse sleep quality (indicated by higher score) than the tinnitus-good sleepers, difference = 4.14, 95% CI [2.56, 5.73] and the controls, difference = 3.44, 95% CI [1.94, 4.94]. The insomnia group scored significantly higher on sleep quality than the tinnitus-good sleepers, difference = 5.18, 95% CI [3.54, 6.82] and the controls, difference = 4.47. 95% CI [2.93, 6.02]. The tinnitus-related insomnia group had better sleep quality than the insomnia group. However, this difference was not significant, difference = −1.03, 95% CI [−2.52, 0.45], nor was the difference between the tinnitus-good sleepers and the controls, difference = −0.71, 95% CI [−2.05, 0.62].

### 3.5. Tinnitus loudness and tinnitus distress between tinnitus-related insomnia and tinnitus-good sleepers’ groups

The tinnitus-related insomnia group had greater tinnitus distress (*M* = 58.54, SD = 23.04) than the tinnitus-good sleepers’ group (*M* = 41.9, SD = 21.62). This difference was significant (16.64, 95% CI [6.91, 26.28]). The tinnitus-related insomnia group experienced significantly louder subjective tinnitus volume than the tinnitus-good sleepers’ group (median difference = 10, *U =* 626, *z =* −2.61, *p* = 0.09). As this was unexpected, *post-hoc* one-way ANCOVA explored whether sleep-related cognitions (DBAS-16) and behaviors (SRBQ) differed between the two groups when controlling for tinnitus loudness (VAS) and distress (THI). The tinnitus-related insomnia group had significantly greater insomnia cognitions, *F*(1, 83) = 35, *p* < 0.001, partial *η*^2^ = 0.3, and behaviors, *F*(1, 83) = 33.1, *p* < 0.001, partial *η*^2^ = 0.29, than tinnitus-good sleepers when controlling for tinnitus distress and loudness, in line with the findings from the main analysis.

## 4. Discussion

In line with the hypotheses, this novel study found that individuals with tinnitus-related insomnia report the same level of dysfunctional sleep-related cognitions and behaviors as individuals with insomnia without tinnitus. For example, catastrophic cognitions about having inadequate sleep, which fuels anxiety and worries, hypervigilance to nighttime wakefulness and daytime sleepiness, and unhelpful behaviors such as spending too long in bed or taking daytime naps. These sleep-related cognitions and behaviors were significantly more prevalent in tinnitus-related insomnia than in people with distressing tinnitus who sleep well and in people with neither insomnia nor distressing tinnitus. Such findings support the claim that cognitive–behavioral processes hypothesized to contribute to the maintenance of insomnia (Harvey, 2002) also maintain insomnia in people with tinnitus. This aligns with recent findings from an RCT which found that CBTi targeting specifically sleep-related behaviors and cognitions (e.g., through time-in-bed restriction, psychoeducation about sleep, anxiety, and worry management) led to large, clinically significant improvements in sleep and tinnitus (Marks et al., 2022). Together, these studies suggest that insomnia in patients with tinnitus may involve the same processes that are found in people who have insomnia without tinnitus. Of course, tinnitus may add an additional layer of complexity and challenge for the patient. However, this similarity across groups indicates how tinnitus and insomnia patients can benefit from existing insomnia therapies.

One possible explanation could have been that anxiety and depression symptoms contributed to the impaired sleep and dysfunctional cognitive–behaviors related to sleep, as such symptoms were significantly higher in both groups with insomnia. However, the fact that the differences in sleep remained after controlling for anxiety and depression demonstrates that sleep-related cognitive–behavioral factors, in fact, explain unique variance in the experience of insomnia both with and without tinnitus.

This study offers new insight into possible maintaining factors for sleep difficulties reported by people with distressing tinnitus. The finding that people with tinnitus-related insomnia reported greater levels of unhelpful sleep-related cognitions, behaviors, and sleep quality than tinnitus-good sleepers indicates that the factors differentiating these two groups relate to insomnia-relevant processes (cognitions and behaviors) rather than tinnitus-relevant processes. Furthermore, there were equivalent levels of sleep-related cognitions, behaviors, and quality in the insomnia-only and tinnitus-related insomnia groups. This adds significant weight to the literature regarding shared cognitive–behavioral characteristics between the two experiences of insomnia, i.e., with and without tinnitus (Crönlein et al., 2016). Considering existing evidence in support of shared biological characteristics between insomnia and tinnitus-related insomnia (Burgos et al., 2005), these findings support a biopsychosocial model of tinnitus-related insomnia.

Unexpectedly, subjective tinnitus volume was louder for those with tinnitus-related insomnia than for those who sleep well, counter to evidence that tinnitus distress is not directly associated with tinnitus volume (Basile et al., 2013). While this may represent a novel difference between people with tinnitus-related insomnia and those without insomnia, further research is needed to draw firm conclusions and explain why this may be the case. The findings from the main analyses did not change when controlling for tinnitus severity and volume, supporting the argument that sleep-related cognitive–behavioral factors maintain tinnitus-related insomnia and countering the hypothesis that tinnitus volume fuels insomnia (Izuhara et al., 2013; Aazah and Moore, 2019).

Another unexpected finding was a lack of significant difference in sleep-related behaviors between those with tinnitus-related insomnia and controls. One possible explanation may relate to the recruitment strategy whereby recruitment of tinnitus groups directly focused on sleep, while recruitment of the control group took a broader approach, which may have led to a tinnitus sample with greater concerns about sleep than the controls.

### 4.1. Strengths

This is the first study that has compared insomnia-related thoughts and behaviors reported by people with tinnitus to people with insomnia, people with tinnitus who sleep well, and individuals without distressing tinnitus or insomnia. The use of multiple comparators is a key strength as it allows for clarification of similarities and differences across all groups and highlights how similar cognitive–behavioral factors in tinnitus-related insomnia are to insomnia without tinnitus. The use of the ISI (Morin, 1993) to assess for the presence of insomnia, which is a validated outcome measure for insomnia research (Bastien et al., 2001), is scarce within tinnitus literature (Asnis et al., 2018) and the robustness of measures collected across the sample is another strength.

### 4.2. Limitations

The possibility of participants having undiagnosed sleep disorders, in addition to insomnia, is a limiting factor in this study. The researchers collected an outcome measure intended to screen for the possibility of undiagnosed sleep disorders (The Sleep Diagnostic Algorithm, Wilson et al., 2010). However, it is briefness meant that no participants were excluded based on responses. Sleep disorders are diagnosed using thorough clinical assessments, which future research in this field should aim to include.

Some participants allocated to the insomnia or control group also reported tinnitus. This is because the allocation of participants to groups was based on participant self-selection of tinnitus distress, rather than score on a tinnitus measure. Interestingly, there was a mismatch, with some self-reported “mild” or “no” tinnitus sufferers meeting criteria for moderate tinnitus on the THI. This could mean that sleep-related cognitions and behaviors measured in the controls and insomnia groups are impacted by the presence of tinnitus. This would limit the extent to which comparisons between tinnitus-related insomnia and insomnia groups can be drawn. Using the THI to sort participants into groups would have avoided this limitation. However, this would require every person in the study to complete the 25-item tinnitus questionnaire (THI). Along with increasing the questionnaire burden, this could cause confusion for participants who say they experience no tinnitus. They would be asked to respond to statements such as “because of your tinnitus do you feel desperate?.” As there are clearly pros and cons to each method of grouping criteria, future studies should consider the limitations of both methods prior to undertaking their study. The study is further limited by the questionnaires not being counterbalanced and solely using self-report measures and cutoffs to classify people as having insomnia, tinnitus, or both. Future studies should consider more robust assessments that are more in line with the clinical diagnostic process for each condition and administered in a randomized way. In addition, the field would benefit from future studies using consistent, standardized measures to compare outcomes more easily.

This study is limited by the sample’s lack of ethnic diversity. The study did not collect any information about participants’ socio-economic status, which may mean we are missing information about the prevalence of tinnitus and insomnia in different fractions of society. A large proportion of the sample reported having further education experience, which can improve cognitive flexibility and lead to better coping strategies. Future studies should set out an *a priori* strategy to recruit a more diverse sample and to set hypotheses around potential differences between groups, such as gender differences (Richter et al., 2021). This will allow the conclusion to be more representative of society and could lead to new insights into risk and protective factors.

## 5. Conclusion and clinical implications

This study demonstrates that insomnia-related cognitive and behavioral processes are very similar in people with insomnia, both with and without associated tinnitus, and that these are different from people with tinnitus who sleep well. The study replicates findings that people with both tinnitus and insomnia report greater anxiety, depression, and tinnitus-related distress than tinnitus sufferers without insomnia (Asnis et al., 2018) but shows that such insomnia-related cognitions and behaviors remain important even when such differences are accounted for. This suggests that difficulties with sleep reported by many tinnitus sufferers can be understood by recognizing that they are engaging in key sleep-related thoughts and behaviors that are stopping them from sleeping, as reported by people with insomnia and explained by the cognitive–behavioral model of insomnia (Harvey, 2002).

Cognitive–behavioral therapy for insomnia has been shown to work with insomnia co-occurring with other physical health problems, such as chronic pain (Jungquist et al., 2010; Tang et al., 2012), and evidence has indicated it is also effective in tinnitus-related insomnia (Marks et al., 2019, 2022). The findings from this study support emerging evidence that people presenting with tinnitus-related insomnia could benefit from treatments already shown to work effectively on people with insomnia disorder, such as CBTi, targeting sleep-related cognitions and behaviors.

## Data availability statement

The raw data supporting the conclusions of this article will be made available by the authors, without undue reservation.

## Ethics statement

The studies involving human participants were reviewed and approved by University of Bath Psychology Ethics. The patients/participants provided their written informed consent to participate in this study.

## Author contributions

EM and GB designed the study. GB collected the data for the study, analyzed the data, and wrote the manuscript. EM contributed substantially to the reviewing and editing of the manuscript and supervised the study. All authors contributed to the review of the article and approved it for submission.

## Funding

This research took place as part of a doctoral undertaking (Barry, 2020), so it was supported by the University of Bath.

## Conflict of interest

The authors declare that the research was conducted in the absence of any commercial or financial relationships that could be construed as a potential conflict of interest.

## Publisher’s note

All claims expressed in this article are solely those of the authors and do not necessarily represent those of their affiliated organizations, or those of the publisher, the editors and the reviewers. Any product that may be evaluated in this article, or claim that may be made by its manufacturer, is not guaranteed or endorsed by the publisher.
