# Supplementary material for: Cognitive–behavioral factors in tinnitus-related insomnia
Source: Front Psychol. 2023 Mar 17;14:983130. doi: 10.3389/fpsyg.2023.983130 (PMC10064054; doi:10.3389/fpsyg.2023.983130)
Supplement: Supplementary file 1 [file Table_1.DOCX]

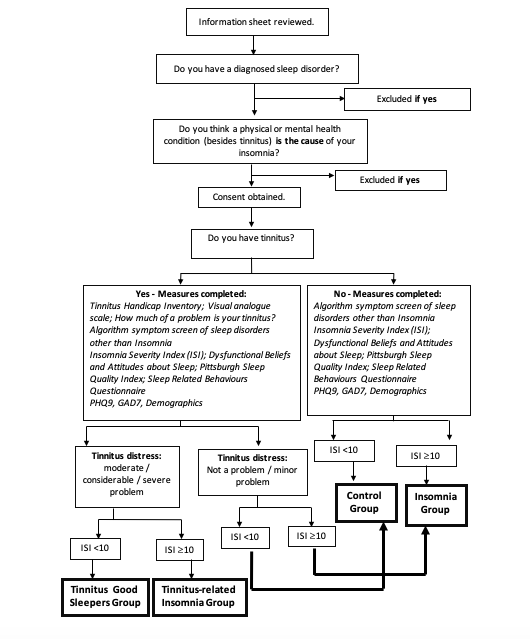
Supplementary Material

**Supplementary Figure 1.** Flow chart for the studies recruitment strategy.
